# Onset and progression of dental erosion in a mouse model

**DOI:** 10.2340/aos.v83.41193

**Published:** 2024-09-09

**Authors:** Julie Marie Haabeth Brox, Amela Tulek, Amer Sehic, Aida Mulic, Tor Paaske Utheim, Qalbi Khan

**Affiliations:** a Department of Oral Biology, Faculty of Dentistry, University of Oslo, Oslo, Norway; b Nordic Institute of Dental Materials (NIOM AS), Oslo, Norway; c Department of Maxillofacial Surgery, Oslo University Hospital, Oslo, Norway; d Department of Clinical Dentistry, Faculty of Health Sciences, UiT The Arctic University of Norway, Tromsø, Norway; e Department of Public Health and Sport Sciences, Inland Norway University of Applied Sciences, Elverum, Norway

**Keywords:** Acidic drinks, Dental enamel, Mouse model, Scanning electron microscopy, Tooth attrition, Tooth erosion

## Abstract

**Objective:**

Purpose of this research was to examine the onset, progression and wear rates of dental erosion in an established mouse model.

**Material and methods:**

Dental erosion in mice was experimentally induced, and the acidic effects of cola drink on their teeth after 2, 4 and 6-weeks were closely analysed by scanning electron microscopy. The tooth height and enamel or dentin loss were established.

**Results:**

The dental erosion on the molars showed clear progression from 2 to 6 weeks. By the 2-week mark, a significant portion of enamel was already eroded, revealing the dentin on the lingual cusps. When adjusted for attritional wear, molars exposed to cola for 2 weeks showed a 35% drop in lingual tooth height compared to controls (533 μm vs. 818 μm). At 4 and 6 weeks, the cola-exposed group continued to display decreased lingual tooth heights by 40% (476 μm vs. 799 μm) and 43% (440 μm vs. 767 μm), respectively.

**Conclusion:**

This study revealed significant acidic effects of cola drink on mouse molars as early as 2 weeks. These findings highlight the challenge of monitoring dental erosion clinically and underscore the importance of early preventive and intervention measures.

## Introduction

Dental erosion, defined as the acid-mediated loss of dental hard tissue, is a complex condition influenced by an array of extrinsic and intrinsic acidic etiologies [1]. Extrinsic acidic beverages and food encompass a diversity of acid types, each of which contributes to the potential risk of dental erosion onset in humans [2–4]. The prevalence of this condition is increasing, mainly because of a change in nutritional habits and lifestyle [5, 6]. Predominantly among adolescents, the intake of acidic beverages has witnessed a notable increase in recent decades, consequently elevating their susceptibility to erosion [4, 7, 8]. Previous research indicates that dental erosion typically begins in the early stages of life and tends to progress as one ages [9, 10]. While the incidence of dental erosion in Norway has remained consistent over the past 30 years, current cases exhibit increased severity [11]. Lifestyle choices and individual drinking habits vary from person to person, influencing the time acid remains in the mouth prior to being swallowed. Nevertheless, clinical observations persistently indicate that dental erosion can manifest or remain absent irrespective of these aforementioned factors. The underlying rationale remains unclear, yet prevailing hypotheses propose that an individual’s predisposition to dental erosion may be modulated by genetic disparities [7, 12, 13], in conjunction with specific oral environmental factors [13, 14].

It is pertinent to note that human *in vivo* experiments, which can induce irreversible damage to dental hard tissues, are deemed ethically untenable. In previously utilised animal models, the applied methodologies were constrained in their ability to capture intricate details and so failed to adequately document minor dental erosion and the associated depth [15, 16]. Therefore, for an extended period, the imperative for a standardised animal model to study dental erosion has been emphasised [17]. A mouse model, tailored for extrinsic dental erosion, wherein lesions of varying severities can be induced and analysed, was established in our recent studies. The current technique, involving transversely ground molars examined via scanning electron microscopy (SEM), facilitates the documentation of dental erosion and its respective depths, even in diminutive dentition such as mouse molars [18]. The model developed in this study provided the opportunity to investigate the salivary contribution to the development of dental erosive wear, demonstrating that mice with reduced salivary flow showed significantly more erosion. Additionally, it was possible to preliminary propose the common pattern of acid-induced dental hard tissue destruction [19]. However, within our model, the accelerated degradation of the tooth surface could also be attributed to the intensified conditions, in which the mice were subjected to a continuous exposure of acidic beverages for a duration of 6 weeks. Moreover, all assessments were executed subsequent to the 6-week experimental duration, making it arduous to ascertain the specific point of time at which the mice began manifesting erosion. Thus, our comprehension of the erosive onset and progression rate is still indeterminate. We further postulated that the tooth wear observed in our research [18, 19] was primarily attributable to erosion rather than attrition. Nonetheless, our findings so far did not provide conclusive evidence to substantiate this theory. The aim of the present study was to further investigate the onset and progression of dental erosion in an established mouse model. To trace and map out a chronological framework for the dental erosion development process and to evaluate possible signs of dentin response to erosive wear. Additionally, by analysing the control group that was administered water *ad libitum*, we aimed to gain a more profound comprehension of attrition’s influence over the same timeline.

## Material and methods

### Animal model

**Figure 1:**
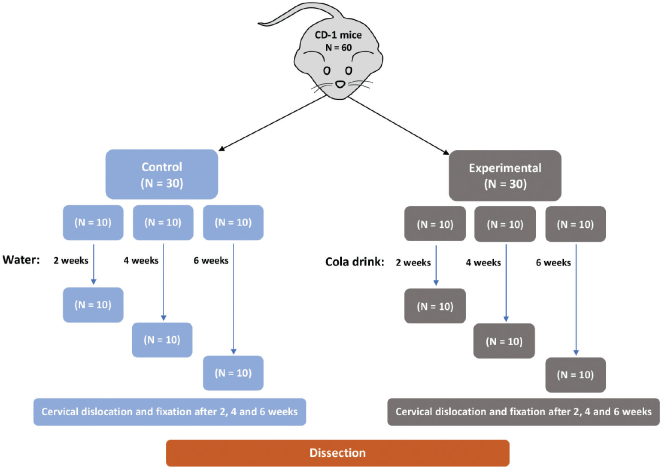
Flow chart of animals enrolled in the study. Sixty CD-1 mice were randomly distributed into two experimental groups, which were provided with cola drink (experimental) and distilled water (control). Each group (*n* = 30 animals) was further divided into triplicate subgroups, that is, 10 animals per cage. After the experimental period of 2-, 4- and 6- weeks, all animals were sacrificed by cervical dislocation.

Figure 1 presents the flow chart of the experimental procedure. Sixty phenotypical, female mice (CD-1 strain, 7 weeks old, 30 ± 5g body wt) were selected. The animals were given standard laboratory fodder and water *ad libitium* prior to experimental use. They were kept under a 12-h light-to-dark cycle at a consistent temperature of 21ºC and a relative humidity level of 65%. The animals were kept in accordance with Norwegian regulation and legislation (Norwegian Regulation on Animal Experimentation of 2015 based on the EU directive on the Protection of Animals used for Scientific Purposes 2010/63/EU and Norwegian Animal Welfare Act of 2009). The experiment was approved by the Norwegian Food Safety Authority (FOTS ID 28734).

Prior to initiating the erosive experiments, wire cages with solid bases and bedding were readied to mitigate dentition wear resulting from attrition. Potential abrasives like wooden sticks and plastic wheels were systematically removed, leaving the animals with paper boxes and paper ribbons for environmental enrichment. The cages were substituted two times per week, and routine daily surveillance of the animals was upheld. Furthermore, to curtail tooth attrition during the investigation, standard laboratory fodder was softened prior to feeding. Fifty units of Teklad Global 18% Protein Rodent Diet (Envigo Teklad, Madison, WI, USA) were immersed in 165 ml of cold tap water. These were then enclosed in a plastic bag and set aside to soften throughout the night.

Sixty mice were randomly distributed into two experimental groups, which were provided with cola drink (Coca cola, phosphoric acid, pH = 2.27) and distilled water (control), respectively. Each group (*n* = 30 animals) was further divided into triplicate subgroups, that is, 10 animals per cage. Two 250 ml bottles with drinks were placed into each cage, and the bottles were replaced three times per week. Prior to the experiment, the changes in pH of cola drink in a 250 ml experimental bottle were monitored over a period of 3 days, and the results showed no changes in pH. During the experiment, all animals were provided with softened laboratory fodder and drinks *ad libitum*, and the consumption of drinks per cage was recorded. After the experimental periods of 2, 4 and 6 weeks, the corresponding subgroups of animals were sacrificed by cervical dislocation, and their heads were fixed in 70% ethanol. All animals were weighted at the start and at the end of the experiment.

### Scanning electron microscopy

The mandibular molars were dissected out and fixed in 70% ethanol. The isolated teeth were thoroughly cleaned by dissection and by gentle brushing under running tap water. The specimens were air dried overnight and mounted on brass cylinders with cyanoacrylate glue, sputter coated with 30 nm platinum and observed in a Philips XL30 ESEM (Philips, FEI, the Netherlands) operated at 12 kV and Tabletop Microscope TM4000Plus (Hitachi, Tokyo, Japan) operated at 10 kV.

The jaw segments containing all three molars were thereafter embedded in Epon and ground transversely. The grinding was performed under a stereo-microscope using grits 800 and 1200 3 M waterproof silicon carbide paper (3 M, St. Paul, MN, USA) in a specially designed apparatus [20]. The ground surfaces were then polished by grinding the specimens against the backside of the 3 M waterproof silicon carbide paper with 0.05 μm particle size alumina powder (Buehler Micropolish, Buehler, Lake Bluff, IL, USA) in water. After careful brushing under running tap water and removal of excess water, the teeth were etched for 45 s in 1% nitric acid, air-dried overnight, sputter-coated with 30 nm platinum and observed in scanning electron microscopy (SEM). The grinding method creating a transversal ground plane for observation in SEM was otherwise as described previously [18].

### Measurements and statistical analysis

SEM images of the transversely ground and etched plane were used for measurements of the lingual tooth height and lingual enamel/dentin loss in distilled water (control) and cola drink mandibular first molars. Mean values and standard deviations were calculated using Microsoft Excel Worksheet (Microsoft Office Excel, 2017). Measurement data were tabulated and analysed using the Statistical Package for Social Sciences 22.0 for Windows (SPSS Inc., Chicago, Illinois, USA). One-way analysis of variance (ANOVA) followed by the Tukey post-hoc test, and independent *t*-tests were used for the evaluation of data. *P*-values < 0.05 were considered statistically significant.

## Results

### Effects of cola drink on onset and progression of dental erosion in mouse molars

**Figure 2:**
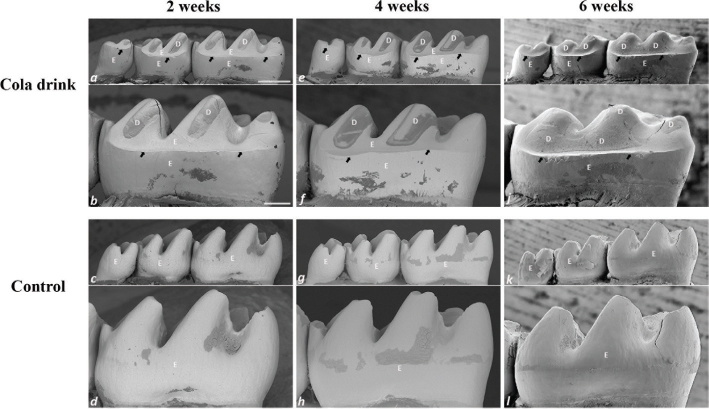
SEM images of lingual aspect of mandibular molars from cola drink (a, b, e, f, i, j) and control (c, d, g, h, k, l) mice. (b, f, j) Higher magnification of lingual view of cola drink mandibular first molar in panels a, e and i, respectively. (d, h, l) Higher magnification of lingual view of control mandibular first molar in panels c, g and k, respectively. The black arrows in panels b, f and j indicate the step between the unaffected cervical part and the affected occlusal part of the cola drink mandibular molars. The bar represents 500 μm in panels a, e, i, c, g and k and 200 μm in panels b, f, j, d, h and l. E = enamel, D = dentin.

**Figure 3:**
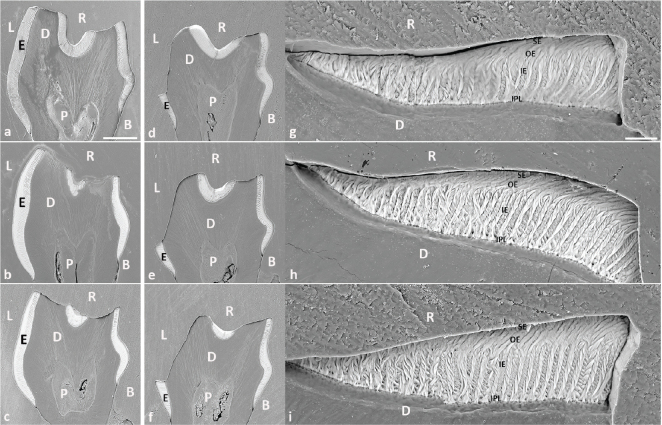
SEM images of transversely ground planes of mandibular first molars from control and cola drink mice. (a, b, c) Transversely ground planes of mandibular first molars from 2-, 4- and 6- weeks control mice, respectively. (d, e, f) Transversely ground planes of mandibular first molars from 2-, 4- and 6- weeks cola drink mice, respectively. (g, h, i) Higher magnification of cervical lingual enamel at the erosive step from d, e and f, respectively. The bar represents 200 μm in panels a-f and 20 μm in panels g-i. E = enamel, D = dentin, P = pulp, R = resin, B = buccal side, L = lingual side, IPL = inner prism-free layer, IE = inner enamel, OE = outer enamel, SE = superficial enamel.

In contrast to the control teeth (Figure 2c, g, k), mandibular molars exposed to cola drinks displayed a rounded cuspal morphology, accompanied by discernible erosion predominantly on the lingual aspects of the teeth (Figure 2a, e, i). A consistent erosion pattern, characterised by a distinct erosive step demarcating the boundary between the unaffected cervical and the affected occlusal regions of the lingual enamel, was present in all cola drink exposed mandibular molars across the various time intervals assessed (Figure 2a, e, i). Intriguingly, as early as 2 weeks, this erosive step, located approximately 240 μm from the enamel-cementum junction (Figures 2b and 3d, g), was continuous across the entire lingual aspect of the first molar. Beyond this step, in the occlusal direction, the enamel was almost entirely eroded on all lingual cusps, leading to exposed dentin (Figures 2b and 3d). The cervical portion of the lingual enamel, residing below the erosive step, remained unaffected and maintained its typical four-layered enamel structure (Figure 3g). After 4 weeks, accelerated enamel loss was clearly manifest (Figure 2f), and erosion of the dentin also became evident, as deduced from the altered lingual contour of the dentinal surface (Figures 2f and 3e). Upon reaching the 6-week point, complete loss of the lingual enamel coupled with significant dentin erosion was observed (Figures 2j and 3f). Yet, of particular interest, the extent of enamel erosion beneath the erosive step did not further advance cervically during the 6-week period, as inferred from the persistent presence of the erosive step (Figure 3d–i).

Through the SEM analysis of both the morphology (Figure 2) and transversal sections (Figure 3) of the mandibular first molars across the three experimental durations (2–6 weeks), the evolving pattern and sequential sequence of dental hard tissue degradation induced by acid during the experimental phase were deciphered. An increasing progression of the dental erosion was evident from the molars at 2 weeks to those at 6 weeks (Figure 2b, f, j). By the 2-week mark, a substantial part of the enamel was already diminished, revealing the dentin on the lingual cusps L1 and L2 (Figures 2b and 3d). The erosive step, which distinguishes the eroded enamel from the untouched cervical enamel, was observable at this interval (Figures 2b and 3d). By the 4-week interval, ongoing erosion had led to an additional diminishment of dental enamel on the L1 and L2 cusps, both in the cervical and mesio-distal directions (Figure 2f). Concurrently, there was an evident escalation in dentin exposure, along with the initiation of its erosion (Figures 2f and 3e). Notably, remnants of enamel still prevailed in the recesses between the cusps, positioned over the erosive step, post both the 2- and 4-week periods (Figure 2b, f). By the 6-week conclusion, nearly the entirety of the lingual enamel situated above the erosive step, excluding a few residual traces lodged in the depressions between the cusps, was eliminated, and the now unshielded dentin manifested as a persistent layer undergoing intensified erosion both cervically, mesio-distally and even in the pulpal direction (Figures 2j and 3f). The profound dentinal erosion towards the pulp, leading to the alteration of the inherent curvature of the lingual dental surface, became unmistakably evident post the 4-week mark (Figures 2f and 3e). This erosive tendency continued, producing a progressively planar dentinal structure by the 6-week culmination (Figures 2j and 3f).

### Erosive versus attritive changes in mouse molars over a 6-week duration

**Figure 4:**
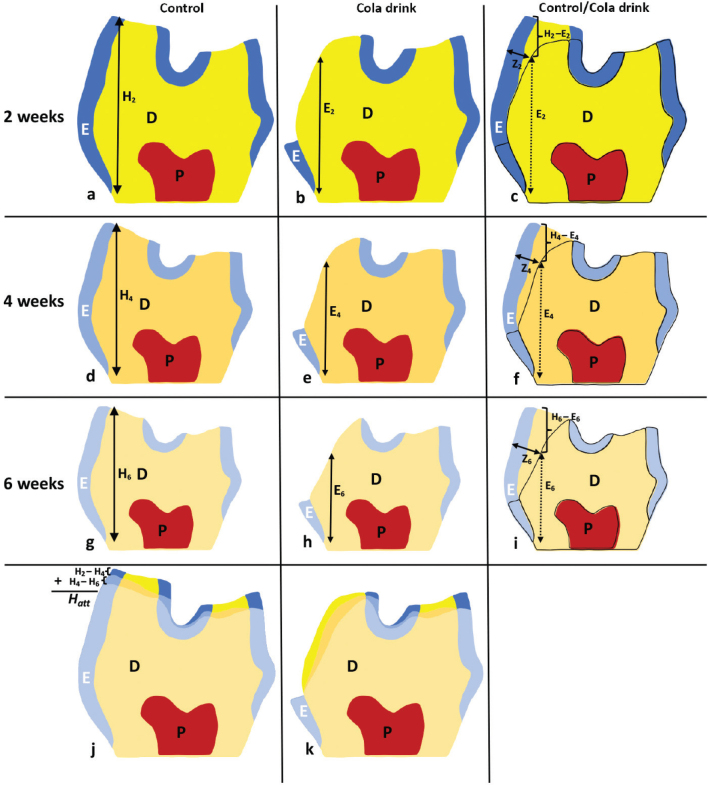
Schematic representation of a transversely ground planes of mandibular first molar from control and cola drink mouse. A separate schematic presentation of transversely ground planes of control (a, d, g) and cola drink (b, e, h) mandibular first molar from 2-, 4- and 6-weeks mice. At the bottom is a collective presentation of ground molar planes from control (j) and cola drink (k) molars over the 6-week period. (c, f, i) A collective presentation of ground molar planes comparing the 2-, 4- and 6-weeks cola drink exposed molars to their control counterparts. H_2-6_ and E_2-6_ indicate lingual tooth height in control and cola drink molars, respectively. Z_2-6_ indicate the loss of the lingual enamel/dentin because of erosion. H_att_ represents total attrition during a 6-week period. Dimensions presented in the figure are correlating with the results in Table 1. E = enamel, D = dentin, P = pulp.

Transversal sections of mandibular first molars reveal differential rates of erosion and attrition in control and cola drink exposed groups over 6 weeks (Figure 4 and Table 1). In an assessment that spanned 2 to 6 weeks, the transversal planes of mandibular first molars from both control and cola-exposed mice were meticulously examined to quantify tooth erosion and attrition rates (Figure 4 and Table 1). A cumulative evaluation of the control group’s molar planes at these timepoints (Figure 4a, d, g) illustrated minimal attritional changes on the lingual cusps, with only about 19 μm attrition from 2 to 4 weeks and an additional 27 μm from 4 to 6 weeks (Figure 4j and Table 1). This results in a total attrition (H_att_)of approximately 46 μm over a 6-week period (Figure 4j and Table 1). In contrast, when comparing the 2-week cola drink exposed molars to their control counterparts (Figure 4a–c), accounting for attrition, a 35% reduction in lingual tooth height (533 μm vs. 818 μm) was observed in the cola drink exposed group. Subsequent evaluations at 4 and 6 weeks revealed that the lingual tooth height remained consistently lower in the cola drink exposed group, by 40% (476 μm vs. 799 μm) and 43% (440 μm vs. 767 μm), respectively, when compared to controls (Figure 4f, i and Table 1).

**Table 1:** Dimensions of lingual tooth height and lingual enamel/dentin loss.

	2 weeks		4 weeks		6 weeks	
	Control	Cola drink	Control	Cola drink	Control	Cola drink
Lingual tooth height	818 ± 23 (H_2_)	533 ± 60 (E_2_)*	799 ± 41 (H_4_)	476 ± 70 (E_4_)*	767 ± 23 (H_6_)	440 ± 36 (E_6_)*
Lingual tooth height loss	285 (H_2_ – E_2_)		323 (H_4_ – E_4_)		327 (H_6_ – E_6_)	
Lingual enamel/dentin loss	65 ± 9 (Z_2_)		98 ± 11 (Z_4_)		118 ± 16 (Z_6_)	
Tooth attrition	19 (H_2_ – H_4_)		27 (H_4_ – H_6_)		46 (H_att_)	

Measured dimensions (mean ± SD, μm) of mandibular first molar lingual tooth height and lingual enamel/dentin loss are presented. The values represent measurements taken from SEM images of transversely ground planes. Dimensions are otherwise as presented in the Figure 4. (*) Significant difference, *p* < 0.05, when compared to control counterparts.

### Dentin response to erosive wear

**Figure 5:**
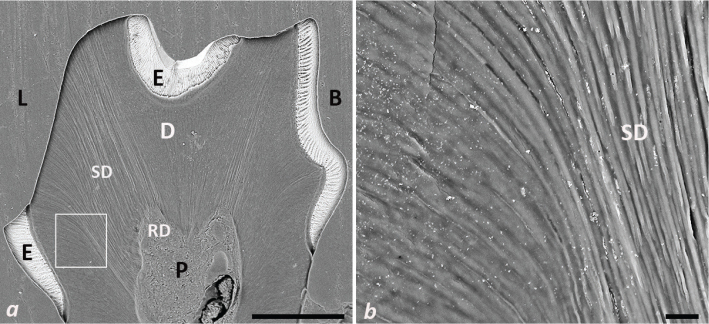
Dentin response to erosive wear. (a) Transversely ground plane of mandibular first molar from 4 weeks cola drink mice. (b) Higher magnification of lingual dentin from marked area in a. The bar represents 200 μm in panel a, and 10 μm in panel b. E = enamel, D = dentin, P = pulp, B = buccal side, L = lingual side, SD = sclerotic dentin, RD = reactionary dentin.

Under typical conditions, the outer terminations of the coronal dentinal tubules are covered by enamel. However, once the dentin becomes exposed to the oral environment, there is a potential for pathways leading to and from the pulp. The analyses revealed a dentinal response in mouse molars to erosive events characterised by the formation of dentin sclerosis and, to some extent, reactionary dentin (Figure 5). The cross-sectional view of the mandibular first molars subjected to cola drinks revealed not only dentin involvement but also a partial pulp sclerosis accompanied by the formation of reactionary dentin. Hence, the erosive wear appeared to stimulate the dentin-pulp-complex to produce reactionary changes here.

## Discussion

Despite the abundance of *in vitro* and *in situ* research emphasising risk indicators and preventive interventions for dental erosion, there is a limited corpus of studies that explore the impact of specific risk indicators associated with dental erosion in animal models [15, 21–24]. In the context of dental research, standardised *in vivo* models present an invaluable tool. In contrast to *in vitro* and *in situ* methodologies that exclude the complexities of saliva and soft tissue interactions, *in vivo* models are particularly poised for advanced studies aimed at elucidating the nuanced roles of salivary dynamics in the genesis of dental erosion. One salient benefit of employing animal models, as opposed to human subjects, lies in the capacity to conduct experiments under heightened control parameters. Additionally, it is pertinent to note that human *in vivo* experiments, which can induce irreversible damage to dental hard tissues, are deemed ethically untenable. Therefore, animal models offer significant merit, primarily because they replicate the interplay of salivary dynamics and soft tissue interactions that mirror the human oral milieu.

Evaluating progression of dental erosion is crucial as it helps establish the need for preventive strategies, assesses the efficacy of interventions and assists in decision-making regarding the timing and methods for restoring eroded teeth. Estimating progression can be achieved through measures such as depth [25, 26], area [27] or volume [28]. However, so far clinical wear rates are not well documented, and erosive wear is hypothesised to not remain constant but may present in spurts. This variability can arise from external influences, such as lifestyle changes (extrinsic factors), or internal risk factors, often associated with episodes of reflux [13, 29]. Furthermore, contemporary societal ideals favoring a lean and fit physique may lead to excessive physical exercise coupled with the consumption of acidic foods and drinks perceived as healthy [30]. Defining a ‘physiological’ wear rate is challenging, not merely because of the lack of a definitive scientific concept. Indications of progression in a clinical setting include a matte surface on the tooth and the lack of extrinsic discolorations. Nonetheless, quantitatively gauging tissue loss can be challenging, given that reference points on the tooth surface can evolve over time. Tracking erosive wear in specific patients by juxtaposing successive high-quality dental molds or images offers a means for visually gauging wear evolution. This method is fundamentally sound for determining treatment strategies, yet its integration into routine dental practice might pose challenges [31].

In our previous studies, the observed pronounced erosive destruction of the tooth surface may be due, in part, to the extreme conditions, where mice had constant access to acidic drinks over 6 weeks [18, 19]. All evaluations took place after this 6-week period, making it challenging to pinpoint the exact time the erosion effects became apparent. As such, the rate of erosive progression along with the point of time where the bulk of enamel may be lost remains unclear. The present investigation offers, for the first time, a deeper insight into how dental erosion develops in a mouse model. The findings revealed a clear escalation in the progression of dental erosion from the 2-week to the 6-week molars. Notably, the most striking observation is that as early as 2 weeks, a considerable amount of the lingual cusp enamel was already eroded, exposing the dentin. Consequently, it is imperative for clinicians to adopt a proactive approach when working with patients exhibiting developmental enamel defects, those experiencing diminished salivary output or individuals frequently consuming foods and beverages with high acidity. This preventive strategy aims to address potential issues before they escalate.

Upon exposure to cola drink, mandibular molars displayed pronounced erosive defects lingually, but the buccal surface remained unaffected (Figure 3). This finding aligns with our prior research [18, 19]. A plausible reason for the difference observed between buccal and lingual aspects might be the short residence time of the acid in the mucobuccal fold, preventing erosion on the buccal side, in contrast to the prolonged exposure in the sublingual region causing lingual erosion. The exact reasons, whether related to rodent chewing dynamics, tongue or muscular actions or other factors, are yet to be elucidated. We also observed a pronounced demarcation in the enamel on the lingual side, marking the boundary between the intact cervical region and the eroded occlusal area (Figures 2 and 3). Notably, while the adjacent enamel and dentin showed significant erosion, this cervical enamel remained unaffected in cola-exposed molars. Similar erosive patterns have been observed in rat [15, 21, 22] and mouse molars [18, 19]. The gingiva shields part of this cervical enamel, which appears to be a protective factor. This prominent observation after just 2 weeks underscores the predominant erosive effect in the cervical direction, indicating that the enamel is not eroded uniformly across the entire lingual surface. It can be hypothesised that gingival crevicular fluid also may play a significant role in this context, either because of its flow dynamics or its pH levels.

Much like *in vivo* enamel erosion, there is limited knowledge regarding the histological aspects of dentin erosion occurring within the oral cavity. It has been established that, similar to caries, dentin exhibits a response to erosion and other wear forms through the development of dentin sclerosis, reactionary dentin and dead tracts [32]. A study analysing teeth from both animals and humans with exposed dentin because of attrition found reactionary dentin in all human permanent teeth (all devoid of caries and restorations) [33]. Moreover, 82% exhibited bacteria within the dentinal tubules, 64% showed inflammation, 29% displayed substantial degenerative changes and 7% evidenced pulpal necrosis. Nonetheless, our understanding of the pulpo-dentinal complex’s response to erosive wear remains limited and even less in regard of both the speed and amount. Given the prevalent exposure of dentin in dental conditions, such as cupped cusps, cervical lesions or gingival recession, there must exist robust defensive mechanisms counteracting bacterial invasion or the diffusion of inflammatory agents through the dentin. From a clinical standpoint, no indications suggest that dentin exposure because of erosive wear can inflict significant pulp damage, provided the rate of wear is counterbalanced by the formation of reactionary dentin. To conclude, the histological profile of eroded dental hard tissues is defined by progressive surface mineral loss, initiating from the natural surface and moving towards the pulp, especially if erosive conditions continue. A notable distinction between the erosion of enamel and dentin is the resistance of dentin’s organic component to clinically relevant acid attacks. Recognising the histology of erosive wear is crucial when formulating preventive, non-invasive and restorative approaches and when setting the parameters for experimental designs.

Observing the enamel loss on the lingual side of a tooth is optimally done using transverse ground sections. Because of the diminutive size of mouse molars, obtaining the perfect transverse plane across the appropriate cusps presents a challenge. This led to minor variations in section positioning and may have contributed to the somewhat elevated standard deviation seen in certain measurements (Table 1). Sections not aligned with our established methodology [18, 19] were excluded. These minor discrepancies are not anticipated to obscure any crucial differences in lingual tooth height and enamel/dentin loss among groups. To counter potential random errors, samples from 10 mice, each represented in biological and technical triplicates, were utilised per group (Figure 1). Tooth size might vary slightly among individual mice. Yet, our prior research indicates such variances are minimal in mice with similar body weights [34–37]. Using the CD-1 strain, with its well-documented molar morphology and size [37], and consistently integrating water as a control, we believe these variables had minimal influence on measurement precision. It is worth noting alternative measurement techniques could have been employed for certain aspects, like lingual tooth height in cola-drinking mice (Figure 4). However, we identified our method as the most consistent and in alignment with control molar measurements.

## Conclusions

This research uniquely demonstrates that the detected hard tissue depletion in mouse molars results from erosion rather than attrition. These insights suggest that tracking dental erosion progression in clinical settings may be challenging, yet it is vital for determining preventive measures and clinical interventions. The potential benefits of various fluoride treatments as preventive measures for those susceptible to dental erosion should also be examined. Future studies might benefit from employing similar animal models and experimental methodologies to address these considerations.
